# Assessment of Pulmonary Toxicity Induced by Inhaled Toner with External Additives

**DOI:** 10.1155/2017/4245309

**Published:** 2017-01-16

**Authors:** Taisuke Tomonaga, Hiroto Izumi, Yukiko Yoshiura, Toshihiko Myojo, Takako Oyabu, Byeong-Woo Lee, Takami Okada, Yunshan Li, Kazuaki Kawai, Toshiaki Higashi, Yasuo Morimoto

**Affiliations:** University of Occupational and Environmental Health, 1-1 Iseigaoka, Yahatanishi, Kitakyushu 807-8555, Japan

## Abstract

We investigated the harmful effects of exposure to a toner with external additives by a long-term inhalation study using rats, examining pulmonary inflammation, oxidative stress, and histopathological changes in the lung. Wistar rats were exposed to a well-dispersed toner (mean of MMAD: 2.1 *μ*m) at three mass concentrations of 1, 4, and 16 mg/m^3^ for 22.5 months, and the rats were sacrificed after 6 months, 12 months, and 22.5 months of exposure. The low and medium concentrations did not induce statistically significant pulmonary inflammation, but the high concentration did, and, in addition, a histopathological examination showed fibrosis in the lung. Although lung tumor was observed in one sample of high exposure for 22.5 months, the cause was not statistically significant. On the other hand, a persistent increase in 8-OHdG was observed in the high exposure group, indicating that DNA damage by oxidative stress with persistent inflammation leads to the formation of tumorigenesis. The results of our studies show that toners with external additives lead to pulmonary inflammation, oxidative stress, and fibrosis only at lung burdens beyond overload. These data suggest that toners with external additives may have low toxicity in the lung.

## 1. Introduction

Toners are used for colored composite materials in printers all over the world, not only in offices but also in homes. The main component of the core toner particle is carbon black, and external additives are used to improve its transfer efficiency. The main components in the additives on the surface of the toner particles are titanium dioxide (TiO_2_) nanoparticles and amorphous silica nanoparticles. Until now, reports on the harmful effect of toner particles have evaluated mainly the core toner itself, but toner particles with the external additive of nanoparticles as a final product have not been investigated in detail [1–6]. It has been reported that nanometer sized particles generally cause greater pulmonary inflammation than micrometer sized particles [7–9]. Ferin et al. [7] performed intratracheal instillation and inhalation studies in rats using TiO_2_ particles of two different sizes and reported that the TiO_2_ particles of a smaller diameter caused a greater pulmonary inflammation at the same mass burden. Li et al. [8] also reported that exposure to different sizes of amorphous silica particles resulted in a size-dependent cytotoxicity in cultured cells. Considering that nanoparticles have stronger pulmonary inflammation and cytotoxicity, the hazards of toner particles might be affected by the addition of external additive nanoparticles.

In lung disorders caused by inhalant dust, infiltration of neutrophils and alveolar macrophages induce pulmonary inflammation, and persistent or progressed inflammation is likely to lead to irreversible fibrosis and eventually cause tumors [10–12]. It is known that materials with high toxicity, such as silica or asbestos, lead to persistent inflammation and cause fibrosis and tumors [13, 14], although some materials lead to persistent inflammation but do not cause tumors [3, 4, 15–17]. Mcconnell et al. [15] performed a long-term (24 months) nose only inhalation study and showed that exposure to artificial fibers such as rock wool and grass wool leads to pulmonary inflammation but not to tumors. Accordingly, due to forming the basis of tumorigenesis through fibrosis, many materials that cause persistent inflammation have the ability of tumorigenesis, but not all of them necessarily cause tumors. Whether a material leads to fibrosis or tumorigenesis will be unknown and uncertain until an actual long-term inhalation study is performed. We previously reported that persistent inflammation was induced by a toner with external additives in a 3-month inhalation study using rats [18]. Building on that research, we developed a long-term inhalation exposure in an animal model in order to examine the tumorigenesis of a toner with external additives.

## 2. Materials and Methods

### 2.1. Toner

The test toner was provided by Fuji Xerox Co., Ltd., Tokyo, Japan, as an experimental toner sample used exclusively for this study, and is not yet commercially available. The toner was synthesized by dispersed toner components in the liquid phase and covered mechanically and electrostatically with TiO_2_ nanoparticles and amorphous silica nanoparticles as the external additives.

### 2.2. Inhalation Exposure

The inhalation system consisted of a dust generator, exposure chambers (volume; 0.57 m^3^), and gas-liquid-solid separators, as described by Tanaka and Akiyama [19]. To obtain a constant concentration, the toner was mixed with fluidizing particles (small glass beads with a diameter of 250 *μ*m) and was prevented from agglutinating a close toner each other. The mixture was fed into a hopper and transported smoothly via a continuous screw feed into a fluidized bed. Dry air flow was blown through the fluidized bed to transport the toner to the exposure chamber while leaving behind the fluidizing particles. The glass beads only remained the fluidized bed because they were heavier than the toner. The toner aerosol concentration in the exposure chamber was measured daily by the isokinetic suction of air though a glass fiber filter beside the chamber.

### 2.3. Animals

Female Wistar rats were purchased from Kyudo Co., Ltd. (Kumamoto, Japan). The rats were divided into 4 groups: high exposure, medium exposure, low exposure, and a control group. The exposure groups were subjected to inhalation exposure for 6 hours a day, 5 days a week, for up to 22.5 months [19]. The age at the start of exposure was 5–7 weeks. The daily average exposure concentrations and standard deviation in the low, medium, and high exposure groups were 1.08 ± 0.54, 4.10 ± 2.12, and 15.89 ± 3.81 mg/m^3^, respectively. The control rats were exposed to clean air only in a chamber of the same size located in the same air-conditioned room. All procedures and animal handling were done according to the guidelines described in the Japanese Guide for the Care and Use of Laboratory Animals as approved by the Animal Care and Use Committee, University of Occupational and Environmental Health, Japan.

### 2.4. Sacrifice

The rats were injected intraperitoneally with a fatal overdose of phenobarbital after 6, 12, or 22.5 months of inhalation. At each time course the control, low-dose, middle-dose and high-dose groups had at least 10 rats, divided into two subgroups of a minimum of 5 animals each for lung tissue analysis. The first subgroup (5 rats) provided bronchoalveolar lavage, which was collected using a physiological saline that was injected through a cannula inserted in the respiratory tract into the right lung while the left lung was clamped. Three to 10 mL (different volumes of lavage fluid were based on the ages of the animals) of physiological saline was infused in the right lung each time and lavage fluid up to 16 mL in total was collected. The left lung was inflated and fixed by 4% paraformaldehyde at 25 cm H_2_O pressure. The lungs of the second subgroup (minimum 5 rats) were homogenized to extract protein and inflated and fixed by 4% paraformaldehyde at 25 cm H_2_O pressure. The right lung tissue was homogenized with a T-PER tissue protein extraction reagent and then centrifuged (1500 ×g for 10 min). The protein concentration of the supernatant was measured by a Pierce BCA Protein Assay Kit (Thermo Scientific Inc., Rockford, IL), using Bovine serum albumin as a standard. The left lungs were evaluated for histopathological changes. The weights of body, liver, kidney, spleen, and brain were determined in all of the groups, while lung weight was determined in the second subgroup only.

### 2.5. Analysis of Inflammatory Cells in BALF with Cytospin

About 10 to 13 mL of BALF from the rats was centrifuged at 400 ×g at 4°C for 15 min. The supernatant was transferred to a new tube and used for measuring the cytokines in the BALF. The pellets were washed by suspension in PMN Buffer (137.9 mM NaCl, 2.7 mM KCl, 8.2 mM Na_2_HPO_4_, 1.5 mM KH_2_PO_4_, and 5.6 mM C_6_H_12_O_6_) and centrifuged at 400 ×g at 4°C for 15 min. After the supernatant was removed, the pellets were resuspended with 1 mL of PMN Buffer. The number of cells in the BALF was counted by Celltac (NIHON Kohden Corporation, Tokyo, Japan), and the cells were splashed on a glass slide using cytospin. After the cells were fixed and stained with Diff-Quik (Sysmex Corporation, Hyogo, Japan), the number of macrophages and neutrophils was counted by microscopic observation.

### 2.6. Measurement of Chemokine of Heme Oxygenase-1 in Lung Tissue and Bronchoalveolar Lavage Fluid

Lung tissue was homogenized with a T-PER tissue protein extraction reagent and then centrifuged (1500 ×g for 10 min). The protein concentration of the supernatant was measured by a Pierce BCA Protein Assay Kit (Thermo Scientific Inc., Rockford, IL), using Bovine serum albumin as a standard. The total protein concentration was adjusted to a final concentration of 500 mg/mL for cytokine-induced neutrophil chemoattractant-1 (CINC-1) and CINC-2, 4000 mg/mL for CINC-3, and 500 mg/mL for heme oxygenase-1 (HO-1). The concentrations of chemokine and HO-1 in the lung tissue were determined by Quantikine Rat CINC-1, CINC-2, CINC-3 (R&D Systems, Minneapolis, MN) (Cat. #RCN100, #RCN200, and #RCN300), and an HO-1 ELISA Kit (ADI-EKS-810A (Enzo Life Science)). These concentrations were also measured in the bronchoalveolar lavage fluid (BALF) supernatant. Myeloperoxidase (MPO), as an attack factor of inflammatory cells, was also measured in the BALF.

### 2.7. Measurements of 8-OHdG in the Lung DNA

The levels of 8-OHdG in the lung DNA were determined according to our previous report [20] with a slight modification. The lung nuclear DNA was extracted from 100 mg of the right upper lobe. A pretreatment filter (EKICRODISC, Acro LC3CR, Nihon Pall Ltd., Tokyo, Japan) was used for filtration of the digested nucleoside solution, and the filtrate was kept at −80°C until just before measurement. A 40 *μ*L aliquot of the filtrate was injected into an HPLC column (Capcell Pak C18 MG II, 3 *μ*m, 4.6 × (100 mm + 150 mm: series-connected), Shiseido Fine Chemicals, Tokyo, Japan) equipped with an electrochemical detector. The flow rate was 0.7 mL/min. The values of 8-OHdG in the DNA were calculated as the number of 8-OHdG per 10^6^ deoxyguanosine (dG).

### 2.8. Histopathology

The lungs, which were inflated and fixed by 4% paraformaldehyde, and trachea were resected from the surrounding tissue. The lung tissue was embedded in paraffin, and 5 *μ*m thick sections were cut from the lobe. The samples were then sectioned and stained with hematoxylin and eosin. The fields from the rat lungs were evaluated as—, none; ±, slight; +, mild; ++ moderate; and +++, marked. Pulmonary fibrosis was evaluated by the Wagner scale [21] (grade 1, normal lung tissue; grade 2, evidence of dust inhalation in centrilobular macrophages; grade 3, minimal interstitial cellular reaction to the dust; grade 4, evidence of early interstitial fibrosis with discrete lesions). Activation of neutrophil inflammation was evaluated in a myeloperoxidase stain using the lung tissue samples of 12-month exposure.

### 2.9. Statistical Analysis

Analysis of variance (ANOVA) and Dunnett's test were applied where appropriate to determine individual differences using a computer statistical package (SPSS, SPSS Inc., Chicago, IL, USA).

## 3. Results

### 3.1. Characterization of Toner

The fundamental characteristics of the bulk toners, dispersed toners in the testing suspension, and toner aerosol in the exposure chamber are summarized (Table 1). The main components of the toner were polyester resin, carbon black, and wax. The total amount of particles smaller than 100 nm in the external additive was 4-5%, and the main nanoparticles were titanium dioxide and amorphous silica. Following the inhalation study, we examined the harmful effect on the lung of the toner with the external additive.

### 3.2. Organ Weight

**Figure 1:**
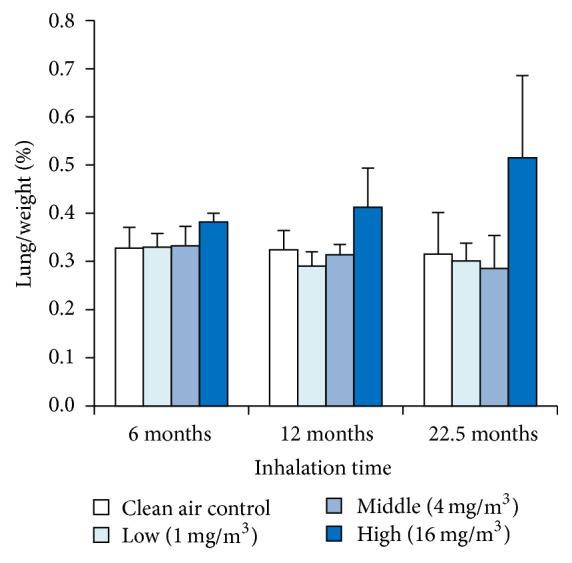
The result of ratio between lung weight and body weight of the rat. Error bar means standard deviation. Asterisks indicate significant differences compared with each control (ANOVA, Dunnett T3).

To evaluate the influence of the inhaled toner over the long-term, we examined the weight of body, lung, liver, kidney, spleen, and brain.  Figure 1 shows the ratio between lung weight and body weight of the rats. Compared with the negative control, there was no significant difference in the ratio between lung weight and body weight of the rats, but a tendency of upregulation was observed in the high exposure groups at all points. On the other hand, since the weight of body, liver, kidney, spleen, and brain showed no significant change compared to the negative control, no influence of toner exposure was observed (data not shown).

### 3.3. Total Cell and Neutrophil Counts in BALF

**Figure 2:**
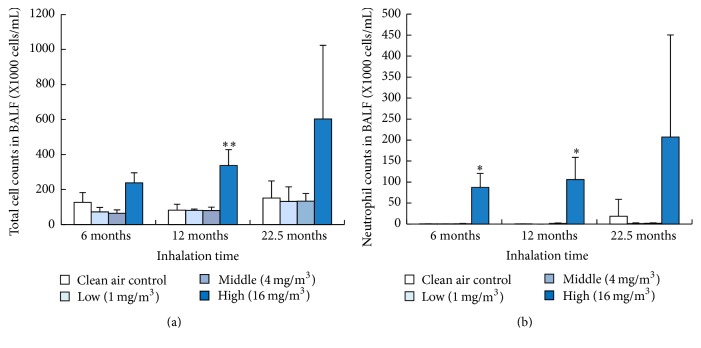
Total cell and neutrophil count in BALF exposed to toner. (a) Total cell count. (b) Neutrophil count. Error bar means standard deviation. Asterisks indicate significant differences compared with each control (ANOVA, Dunnett T3) (^*∗*^*p* < 0.05, ^*∗∗*^*p* < 0.01).

We measured inflammatory cells in BALF to investigate whether the toner induced persistent inflammation (Figure 2).  Figure 2(a) shows the total number of cell counts in the BALF. In the high exposure group, the total number of cells showed a statistically significant increase in 12-month exposure compared to the control.  Figure 2(b) shows the number of neutrophils in the BALF. There was a statistically significant increase in the number of neutrophils in the high exposure group at 6-month and 12-month exposure. A tendency of upregulation was observed in 22.5-month exposure, but it was not statistically significant.

### 3.4. CINC Concentration in Lung Tissue and BALF

**Figure 3:**
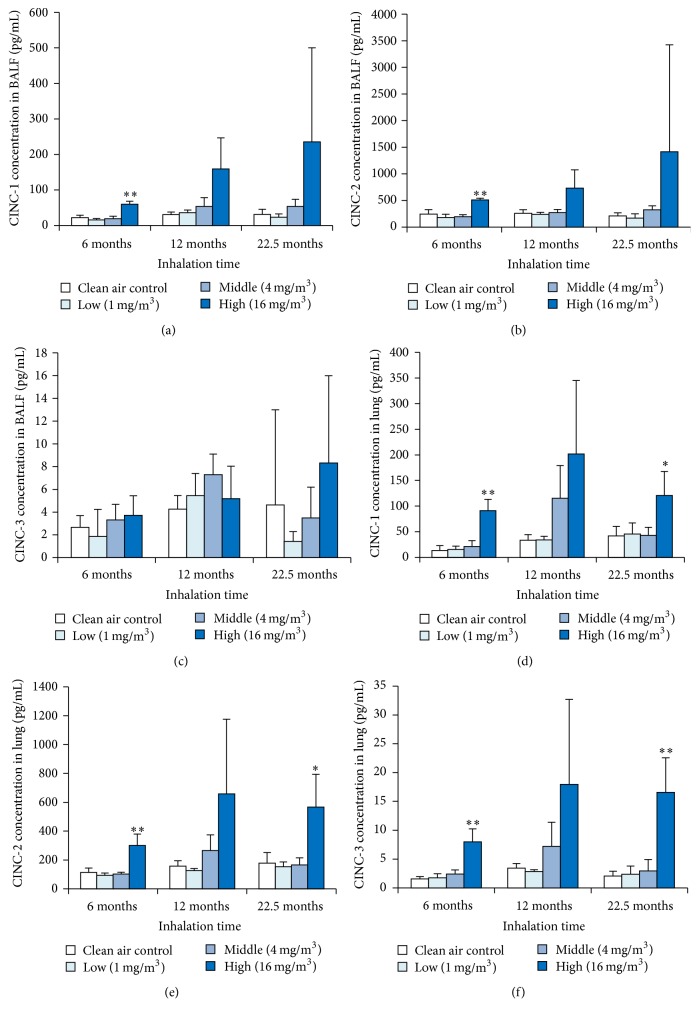
Concentration of CINC in rat lungs exposed to toner. (a) CINC-1 in BALF. (b) CINC-2 in BALF. (c) CINC-3 in BALF. (d) CINC-1 in lung tissue. (e) CINC-2 in lung tissue. (f) CINC-3 in lung tissue. Error bar means standard deviation. Asterisks indicate significant differences compared with each control (ANOVA, Dunnett T3) (^*∗*^*p* < 0.05, ^*∗∗*^*p* < 0.01).

We investigated the chemokines in the BALF and lung tissue (Figures 3(a)–3(f)). There was a statistically significant increase in the concentrations of CINC-1 and CINC-2 in the BALF in the high exposure group at 6-month exposure. Although the concentration of CINC-1 and CINC-2 in the BALF in the high exposure group at 12-month and 22.5-month exposure did not show a statistically significant increase, a tendency of increase was observed. The concentration of CINC-3 in the BALF did not increase significantly, even in the high exposure group, and it did not increase in a dose-dependent manner. In the lung tissue in the high exposure group, the concentrations of CINC-1, CINC-2, and CINC-3 at 6-month and 22.5-month exposure showed a statistically significant increase. Although there was not a statistically significant increase in the lung tissue in the concentration of CINC-1, CINC-2, and CINC-3 at 12-month exposure, a tendency of increase was observed.

### 3.5. HO-1 Concentration in Lung Tissue and BALF

**Figure 4:**
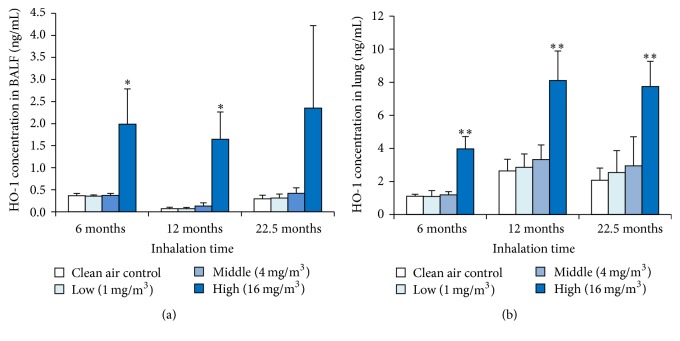
Concentration of HO-1 in rat lungs exposed to toner. (a) HO-1 in lung tissue. (b) HO-1 in BALF. Error bar means standard deviation. Asterisks indicate significant differences compared with each control (ANOVA, Dunnett T3) (^*∗*^*p* < 0.05, ^*∗∗*^*p* < 0.01).

We measured HO-1 in the BALF and the lung tissue to evaluate oxidative stress with the toner (Figure 4).  Figure 4(a) shows statistically significant increases in the concentration of HO-1 in the BALF at 6-month and 12-month exposure.  Figure 4(b) shows statistically significant increases in the concentration of HO-1 in the lung tissue until the end of exposure.

### 3.6. MPO Protein Concentration in BALF

**Figure 5:**
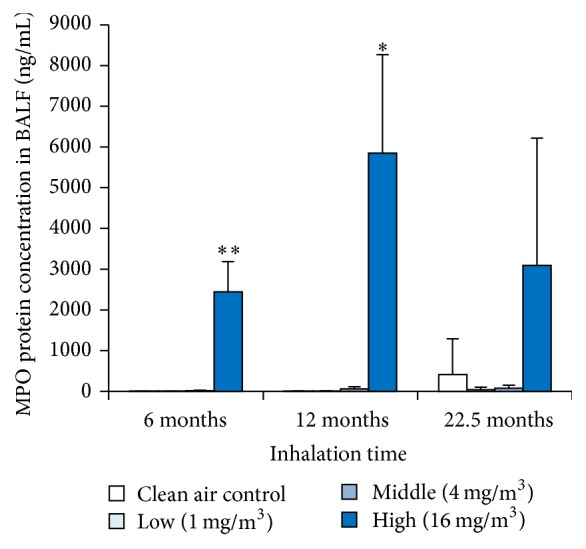
Concentration of MPO in BALF exposed to toner. Error bar means standard deviation. Asterisks indicate significant differences compared with each control (ANOVA, Dunnett T3) (^*∗*^*p* < 0.05, ^*∗∗*^*p* < 0.01).

The concentration of MPO protein in the BALF showed statistically significant increase in the high exposure group at 6 months and 12 months (Figure 5). In the high exposure group at 22.5 months, the concentration of MPO was not significantly higher, although the change was greater than in the other exposure groups.

### 3.7. Pathological Findings

**Figure 6:**
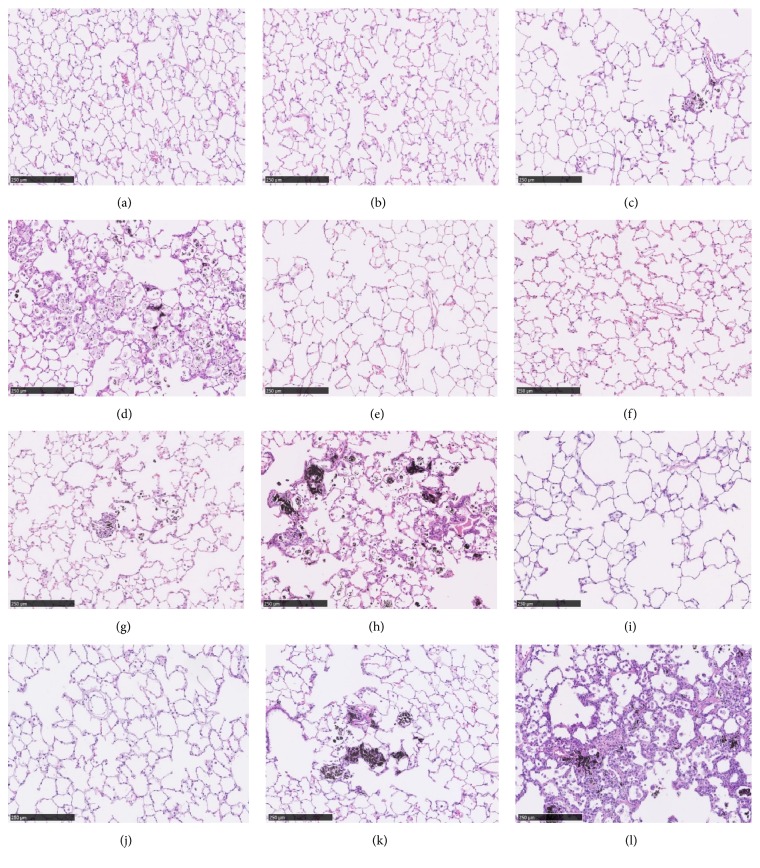
Hematoxylin and eosin staining of lung sections after inhalation of toner. Magnification ×100. (a) Lung of negative control at 6 months. (b) 1 mg/m^3^ toner-exposed lung at 6 months. (c) 4 mg/m^3^ toner-exposed lung at 6 months. (d) 16 mg/m^3^ toner-exposed lung at 6 months. (e) Lung of negative control at 12 months. (f) 1 mg/m^3^ toner-exposed lung at 12 months. (g) 4 mg/m^3^ toner-exposed lung at 12 months. (h) 16 mg/m^3^ toner-exposed lung at 12 months. (i) Lung of negative control at 22.5 months. (j) 1 mg/m^3^ toner-exposed lung at 22.5 months. (k) 4 mg/m^3^ toner-exposed lung at 22.5 months. (l) 16 mg/m^3^ toner-exposed lung at 22.5 months. Multifocal very slight (minimal) to severe bronchioloalveolar hyperplasia and fibrosis was found in lung tissue in every high exposure group.

We investigated the pathological findings by hematoxylin and eosin staining in the lung exposed to toner with external additives (Figures 6(a)–6(l)). Pathological findings in the control and low exposure group at 6 months showed no toner particle-laden alveolar macrophages. Although there was no increase in alveolar macrophages in the middle exposure group at 6 months, similar to the low exposure group, some toner particle-laden alveolar macrophages were observed. In the high exposure group at 6 months, an accumulation of alveolar macrophages was observed in the alveoli, and some of them contained the toner, and mild hypertrophy/hyperplasia of the alveolar epithelium was also observed. In the middle exposure group at 12-month exposure, an accumulation of toner particle-laden alveolar macrophages and transient, mild hyperplasia/fibrosis of the bronchoalveolar epithelium was observed. In the high exposure group at 12 months, mild to severe hyperplasia/fibrosis of the bronchoalveolar epithelium was observed. The results of pathological findings at 22.5-month exposure showed persistently mild to severe hyperplasia/fibrosis of the bronchoalveolar epithelium in the high exposure group but not in the middle exposure group.

**Figure 7:**
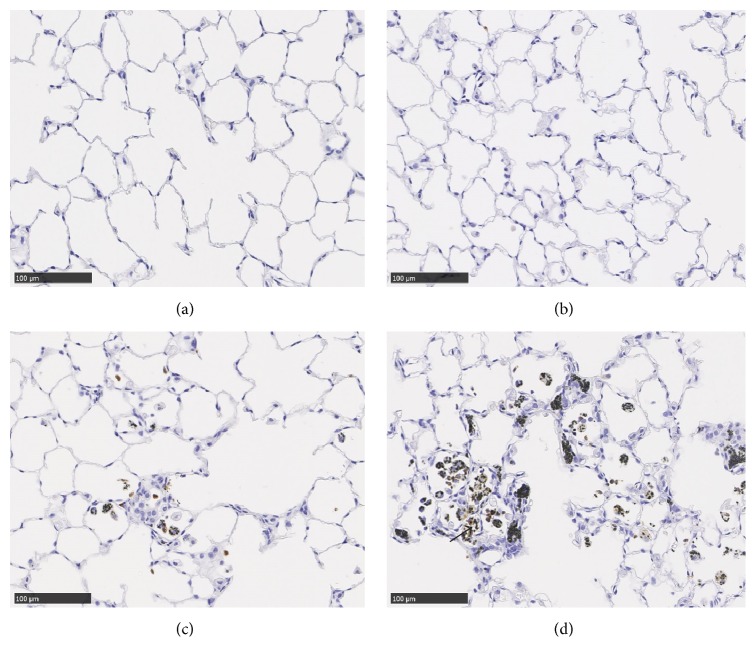
Myeloperoxidase stain of lung sections at 12-month exposure. (a) Lung of negative control. (b) 1 mg/m^3^ toner-exposed lung. (c) 4 mg/m^3^ toner-exposed lung. (d) 16 mg/m^3^ toner-exposed lung.

We used the Wagner scale, in which grade 4 means evidence of early interstitial fibrosis with discrete lesions, to evaluate pulmonary fibrosis. Grade 4 was observed in the high exposure groups in 8 rats at 12-month exposure and in 4 rats at 22.5 months, but not in any rats at 6 months. Grade 4 was not observed in the control, low, and middle exposure groups at any time. These pathological findings in the rat lung are summarized (Table 2). Although there was a lung tumor in only one sample in the high exposure group at 22.5 months, no tumors were significantly caused in a dose-dependent manner. The activation of neutrophils inducing lung injury was evaluated in a myeloperoxidase (MPO) stain using lung tissue samples from 12-month exposure (Figures 7(a)–7(d)). In the high exposure group, positive MPO stain was observed in inflammatory cells around the area of the toner particle-laden alveolar macrophages (Figure 7(d) arrow). A slight positive MPO stain was observed in the middle exposure group, and a negative MPO stain was observed in the control and low exposure groups.

### 3.8. 8-OHdG Levels in the Lung DNA

**Figure 8:**
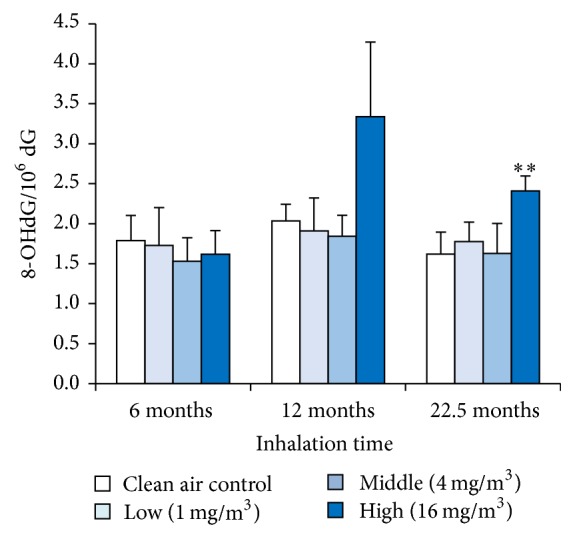
8-OHdG levels in lung DNA after exposure to toner. Error bar represents standard deviation. Asterisks indicate significant differences compared with the control (^*∗∗*^*p* < 0.01).

The 8-OHdG levels in the lung nuclear DNA were significantly higher in the high exposure groups at 22.5-month exposure, while there was no significant increase in the low and middle concentration groups (Figure 8).

## 4. Discussion

We performed inhalation exposure in rats using a toner with external additives at three exposure concentration levels: 1 mg/m^3^ (low); 4 mg/m^3^ (middle); and 16 mg/m^3^ (high). Based on a MPPD 2 model, we calculated that the amount of toner deposited in the rat lung after inhalation exposure (MMAD (GSD) 2.1 *μ*m (1.6), exposure time 6 hours/day, 5 days/week for 22.5 months), in the low, middle, and high exposure levels would be 0.15 mg, 1.06 mg, and 10.6 mg, respectively. Considering this estimated amount, the threshold of overload was suspected to be a dose between the middle and high concentrations. Bellmann et al. [2] reported that 0.4 mg of deposited toner (MMAD (GSD) 4.0 *μ*m (1.5)) (10 mg/m^3^) which was composed of 90% styrene/l-butyl methacrylate random copolymer and 10% high-purity furnace-type carbon black did not change the clearance of toner in rat lung, although 3 mg (40 mg/m^3^) did delay the clearance. Additionally, Morrow et al. [1] made a prediction that the clearance delay of toner and overload in rat lung would be caused when the amount of toner deposit was more than 1 mg. In the present study, the pathological findings in the high exposure group showed significant aggregated toner phagocytized macrophages compared with the low and middle exposure groups. These changes indicated that the amount of toner in the high exposure group reached overload. We previously conducted a 3-month inhalation study using the toner with external additives and the result speculated that the threshold of overload was between 4 mg/m^3^ and 16 mg/m^3^ and that potential exposure level was 1 mg/m^3^ [18]. The present toner with external additives was the same as a past toner. Considering from the past study, we set the concentration of 1 mg/m^3^ as a potential exposure level and 16 mg/m^3^ as an overload level. We estimated the toner with external additives exposed to workers handling a toner. The deposited mass of toner was speculated using the following formula

(1) Deposited mass of toner=exposure concentration of particle×tidal volume×breathing frequency×exposure hours in day×particle deposition fraction.

We calculated the deposition fraction of rat and human for the test toner would be 0.036 and 0.159, respectively (calculated by MPPD 2 model: MMAD (GSD) 2.1 *μ*m (1.6)). We assumed that the toner was deposited at same rate (amount of deposited toner/1 g of lung weight) in rat and human using MPPD 2 model (rat and human lung weight are 1.5 and 1100 g) (calculation in rat and human under assumption of tidal volume; 2.1 and 625 mL/times, breathing frequency volume; 102 and 12 times/min, exposure hours in day; 6 hours), and estimated exposure time per human was 9286 days, if the deposited mass of toner exposed to rats at a concentration of 16 mg/m^3^ for 22.5 months was converted into a human (working time; 8 hours/day, 5 days/week) at a exposure of concentration of 3 mg/m^3^ defined as the threshold limit values-time weighted average (TLV-TWA) of respirable dust by American Conference of Governmental Industrial Hygienists (ACGIH). We think the deposited mass exposed to rats at 22.5 months in the 16 mg/m^3^ toner group may correspond to approximately 36 years of exposure for human at a concentration of 3 mg/m^3^.

In this study, a slight infiltration of macrophages was induced in the low and middle level groups, but there was no persistent pulmonary inflammation. We previously conducted a 2-year inhalation exposure study using ground toner (MMAD (GSD) 3.0 *μ*m (1.7), exposure concentrations were 1.6 mg/m^3^, 4.4 mg/m^3^, and 16.3 mg/m^3^) and polymerized toner (MMAD 4.5 *μ*m, exposure concentrations were 5.5 mg/m^3^ and 15.2 mg/m^3^) and found no persistent pulmonary inflammation when animals were exposed to similar amounts of these toners [3, 4]. Compared with the pulmonary inflammation in previous reports, we predicted that there would be no marked difference in the pulmonary inflammation even if there were differences like a mixture with an external additive or in the composition of the toner due to technical improvements. We previously conducted a 13-week inhalation exposure study using a toner with external additives as same toner in this experiment, titanium dioxide (TiO_2_) particles as a negative control (MMAD (GSD) 1.1 *μ*m (1.6), exposure concentration was 5 mg/m^3^), which composed 99.5% of the rutile, and the* quartz* sample (quartz DQ12) (MMAD (GSD) 1.4 *μ*m (1.8), exposure concentration was 1 mg/m^3^) as a positive control, which composed a crystallinity of 87% a quartz; 13% was amorphous. [18]. Considering that same mass concentration of these materials, the present toner with the external additive may have lower toxicity than quartz DQ12 as a positive control and may be similar toxicity with TiO2 as a negative control.

In the present study, we investigated the mechanism of persistent inflammation by the toner by measuring the concentrations of CINC 1, 2, and 3, representative neutrophil chemotactic factors, and myeloperoxidase in the BALF and in the lung tissue. Results showed that CINC-1, CINC-2, and CINC-3 were upregulated only in the high exposure groups and that these patterns were similar to persistent inflammation. Several studies have previously reported a relationship between the CINC family and pulmonary inflammation. Inhalation of materials with a high potential for inflammation, like nickel oxide nanoparticles [22, 23] and diesel particles [24], showed persistent increases in CINC-1 or CINC-2 expression in the lung in intratracheal instillation studies. On the other hand, TiO2 (micron-size) and fullerene, which are less inflammogenic to the lung, revealed a mild and transient increase in CINC-1 and CINC-2*αβ* expression only in the acute phase in intratracheal instillation studies [23, 25]. In the present study, persistent pulmonary inflammation also induced the upregulation of the CINC family, so we think that interpulmonary infiltration of inflammatory cells including neutrophils is related to the CINC family. The concentration of MPO in high exposure group showed a tendency to increase, and the number of neutrophils in the BALF showed the same tendency. Knaapen et al. [26] reported that an increase in neutrophils induced an increase of MPO concentration in an intratracheal instillation rat model using crystalline silica (DQ12). In the present study, not only an increase in neutrophils but also the upregulation of CINC expression was observed, suggesting that the increase in MPO concentration was related to the activation of neutrophils infiltrated into the lung. Stringer et al. [27] reported that cigarette particles caused the upregulation of CINC-1 expression and activation of MPO in a rat exposure model, and this supports the view that pulmonary inflammation is induced by both the CINC family and MPO. To note, in some subjects in the high exposure group, the upregulation of the CINC family showed trends but not statistical significance. These results may have been affected by individual differences.

In this long-term inhalation study, the toner with external additives did not cause significant lung tumor. There are some long-term inhalation studies similar to the present one [1–4, 28], and none of them showed tumorigenesis in the same kind of long-term inhalation exposure as we performed in this study. Slesinski and Turnbull [28] performed a 104-week inhalation exposure of rats using a non-carbon-based magnetite photocopying toner (MMAD 5.1~5.8 *μ*m) and reported that the toner induced no tumor. This toner contained 45% to 50% magnetite, with 45% to 50% styrene acrylic resin and less than 5% external additives, including hydrophobic amorphous silica and proprietary surface functional modifiers. From the previous report, it is indicated that there would be no marked difference of tumorigenesis even if there were differences in the main component of the toner. On the other hand, Mohr et al. [29] intratracheally injected a high-dose of toner (60–120 mg) into rats and induced lung tumor. One cause of these differences of tumor morbidity may be the difference of deposition amount in the lung.

Although the toner with external additives induced no tumor significantly, we examined 8-OHdG, which is a typical oxidative DNA damage marker, and HO-1, which is an oxidative stress marker, as tumor related factors. Persistent increases in expression of 8-OHdG and HO-1 were observed in the high exposure group up to 22.5 months. It is known that HO-1 is induced by reactive oxygen species (ROS) to protect the cells from ROS production, and there are reports that HO-1 expression is increased by asbestos or silica, which possess tumorigenicity [30–32]. In the present study, the high exposure groups indicated a persistent oxidative stress condition. Yamaguchi et al. [33] reported that 8-OHdG increased in lung tissue when rats and hamsters were intratracheally injected with asbestos, which induced tumor. In the high exposure group in the present study, a persistent increase in 8-OHdG indicated DNA damage by oxidative stress with persistent inflammation, in other words, the foundation of tumorigenesis. However, since these results were caused only in the high exposure group with an excessive amount of deposited toner in rat lung, it is thought that they were influenced by overload.

In conclusion, our long-term inhalation exposure study using toner with external additives for 22.5 months showed that middle level transiently induced slight inflammation and fibrosis, but a high concentration of CINC1-3 and MPO that caused persistent inflammation and fibrosis was induced only in the high level concentration of toner. Only a high concentration of toner induced an upregulation of HO-1 gene expression and 8-OHdG oxidative stress marker, and none of the exposure groups showed tumor in significance. Because persistent inflammation and oxidative stress were observed only in the high exposure group, we speculate that these changes were influenced more by the overload itself than by the toner with external additives. These data suggest that toner with external additives may not have a high potential to cause lung tumor.

## Figures and Tables

**Table 1:** Physicochemical properties of toner.

Test sample	Physicochemical properties	Value
Bulk toner^*∗*^	Color	Black
	Component (wt%)	
	Polyester resin	70–80%
	Carbon black	1–10%
	Wax	1–10%
	Copper compound	1–10%
	Total amount of particles less than 100 nm in external additive (wt%)	4-5%
	Total amount of titanium dioxide nanoparticles in external additive (wt%)	1-2%
	Total amount of amorphous silica nanoparticles in external additive (wt%)	3-4%
	BET surface area^*∗∗*^ (m^2^/g)	2-3
Toner for inhalation study	Mass median aerodynamic diameter (*μ*m)	2.1
	Geometric standard deviation	1.6

Toner^*∗*^: powder used in laser printers and photocopies to form the printed text and images on the paper.

BET surface area^*∗∗*^: Brunauer-Emmett-Teller surface area.

**Table 2:** Pathological finding of lung inhaled toner.

Pathological feature	6 months				12 months				22.5 months			
	Negative control	1 mg/m^3^	4 mg/m^3^	16 mg/m^3^	Negative control	1 mg/m^3^	4 mg/m^3^	16 mg/m^3^	Negative control	1 mg/m^3^	4 mg/m^3^	16 mg/m^3^
	*n* = 11	*n* = 10	*n* = 10	*n* = 11	*n* = 10	*n* = 10	*n* = 10	*n* = 10	*n* = 14	*n* = 13	*n* = 18	*n* = 11
Infiltration in alveolar macrophages	±	±	+	++ ~ +++	±	±	+	++ ~ +++	±	±	+	+++
Hyperplasia of epithelial cell	—	—	—	±	—	—	— ~ +	+ ~ +++	—	—	—	± ~ +++
Fibrosis	—	—	—	±	—	—	— ~ +	+ ~ ++	—	—	—	± ~ ++
Interstitial infiltration	—	—	—	—	—	—	—	—	—	—	—	—
Tumor incidence rate % (count)	0 (0)	0 (0)	0 (0)	0 (0)	0 (0)	0 (0)	0 (0)	0 (0)	0 (0)	0 (0)	0 (0)	9.1 (1)

Grade of changes: — none, ± minimum, + mild, ++ moderate, and +++ remarked.
